# Functional Characterization of BbroAFP Reveals Its Pleiotropic Antifungal Activity in *Botrytis cinerea*

**DOI:** 10.3390/jof12050305

**Published:** 2026-04-23

**Authors:** Arda Örçen, Yunus Doğan, Amjad Tulimat, Beyza Goncu, Batu Erman, Günseli Bayram Akçapınar

**Affiliations:** 1 Department of Medical Biotechnology, Graduate School of Health Sciences, Acıbadem Mehmet Ali Aydınlar University, Istanbul 34752, Türkiye; arda.orcen@live.acibadem.edu.tr; 2 Nanomik Biyoteknoloji A.Ş., Istanbul 34400, Türkiye; yunusdogan@nanomik-tech.com (Y.D.); amjadtulimat@nanomik-tech.com (A.T.); 3 Department of Medical Services and Techniques, Bezmialem Vakif University, Istanbul 34093, Türkiye; bsgoncu@gmail.com; 4 Department of Molecular Biology and Genetics, Faculty of Engineering and Natural Sciences, Acıbadem Mehmet Ali Aydınlar University, Istanbul 34752, Türkiye; batuerman@gmail.com; 5 Department of Molecular Biology and Genetics, Graduate School of Natural and Applied Sciences, Acıbadem Mehmet Ali Aydınlar University, Istanbul 34752, Türkiye

**Keywords:** antifungal protein, *Beauveria brongniartii*, *Botrytis cinerea*, *Pichia pastoris*, cysteine-rich peptide, metacaspase, biofungicide

## Abstract

Fungal pathogens pose a major threat to global agriculture and human health, necessitating alternative antifungal strategies with high efficacy and low resistance potential. Antifungal proteins (AFPs) from filamentous fungi are promising candidates due to their stability, selectivity, and diverse mechanisms of action. Here, we characterize *Beauveria brongniartii* antifungal protein (BbroAFP), a novel cysteine-rich protein from the entomopathogenic fungus *B. brongniartii*, and investigate its antifungal activity against *Botrytis cinerea*. Recombinant BbroAFP was expressed in *Pichia pastoris*, purified, and verified by liquid chromatography–tandem mass spectroscopy (LC–MS/MS) and in silico modeling. BbroAFP showed potent antifungal activity with minimum inhibitory concentrations (MICs) as low as 1 µM against several phytopathogenic fungi, while exhibiting no significant antibacterial activity. Activity was maintained across a wide range of pH and temperature conditions. Confocal microscopy revealed rapid surface binding followed by cytosolic internalization without major cell wall disruption. BbroAFP induced a rapid, transient burst of reactive oxygen species (ROS), accompanied by nuclear DNA fragmentation. Gene expression analysis revealed a transient increase in *aif1*, whereas *mca1* expression decreased at later time points and *mca2* remained largely unchanged, suggesting a metacaspase-independent response. Detached tomato leaf assays showed effective protection against *B. cinerea* without detectable phytotoxicity. Cytotoxicity assays confirmed a favorable safety profile, supporting further evaluation of BbroAFP for plant protection.

## 1. Introduction

Fungal pathogens pose a serious threat to both global agriculture and human health. In agriculture, plant pathogenic fungi are responsible for substantial yield losses in major crops, with annual reductions estimated at 14–18%, corresponding to global economic losses of approximately US$220–300 billion. Among these pathogens, filamentous fungi such as *Botrytis cinerea* are major contributors to crop damage and post-harvest losses [1].

In parallel, fungal infections also represent a major challenge in clinical medicine. Invasive fungal diseases are estimated to cause approximately 1.5–1.7 million deaths worldwide each year, with most fatalities associated with infections caused by *Aspergillus* and *Candida* species [2]. The limited availability of antifungal drugs, together with the increasing emergence of resistant strains, further complicates disease management.

Taken together, the growing impact of fungal pathogens across agricultural and clinical settings highlights the urgent need for alternative antifungal strategies that combine high efficacy with a low risk of resistance development and improved safety profiles.

Antifungal proteins and peptides (AFPs) produced by filamentous fungi are increasingly recognized as promising candidates in this context. These molecules are typically small, cationic, and cysteine-rich proteins stabilized by multiple disulfide bonds, which confer high structural stability and resistance to proteolytic degradation [3,4]. Importantly, their antifungal activity often involves multiple biological processes, including interactions with the fungal cell surface or membrane, induction of cell wall stress responses, reactive oxygen species (ROS) accumulation, and regulated cell death (RCD)-like processes [5,6]. This multifaceted mode of action may reduce the likelihood of resistance development compared with classical single-target antifungal agents.

Previous studies have shown that AFP-induced growth inhibition is frequently associated with the activation of RCD mechanisms, including apoptosis-like processes, autophagy, and intracellular ROS accumulation [7]. Notably, some AFPs can enter fungal cells without causing immediate membrane lysis and subsequently trigger intracellular stress responses leading to cell death. These observations indicate that antifungal activity is not solely based on membrane disruption but also involves intracellular signaling and regulatory pathways.

Antifungal proteins derived from entomopathogenic fungi represent a relatively underexplored source of bioactive molecules. As these organisms coexist with diverse microbial competitors in their ecological niches, the proteins and peptides they secrete may exhibit distinctive antifungal properties. Indeed, antifungal peptides identified from the entomopathogenic fungus *Beauveria bassiana* highlight the potential of this group as a valuable source of antifungal biomolecules [8].

Therefore, the primary objective of this study was to identify and characterize a previously undescribed antifungal protein, BbroAFP, from *Beauveria brongniartii*. In addition to its biochemical characterization, we evaluated its antifungal activity against the plant pathogen *B. cinerea*. To further elucidate its biological properties, in silico structural analyses were performed to predict structural features and potential dimerization behavior. Furthermore, cellular localization experiments, oxidative stress assays, and gene expression analyses were conducted to explore the mechanisms underlying BbroAFP-mediated antifungal activity and to assess its potential relevance for plant disease control.

## 2. Materials and Methods

### 2.1. Strains, Media, and Culture Conditions

The gene encoding the mature BbroAFP peptide (NCBI GenBank Accession No. OAA34374.1) was identified from the *B. brongniartii* genome, codon-optimized for *Pichia pastoris*, and synthesized. The coding sequence was cloned into the pPICZαA vector (Invitrogen, Waltham, MA, USA) under the *AOX1* promoter, in-frame with the *Saccharomyces cerevisiae* α-mating factor secretion signal. A C-terminal 6 × His tag was included for immobilized metal affinity chromatography (IMAC) purification [9].

The recombinant plasmid was linearized and transformed into *P. pastoris* KM71H via electroporation [10]. Transformants were selected on yeast extract peptone dextrose (YPD) agar containing Zeocin (100 μg/mL). Integration was confirmed by colony PCR using *AOX1*-specific primers *aox1* F 5′ GACTGGTTCCAATTGACAAGC 3′ and R 5′ GCAAATGGCATTCTGACATCC 3′ flanking primers.

Positive clones were screened for protein expression, and the best-performing clone was cultured in buffered glycerol-complex medium (BMGY) at 30 °C (final OD_600_ ≈ 30). Expression was induced in buffered minimal methanol (BMM) with daily addition of 1% (*v*/*v*) methanol for 72 h at 30 °C [11].

### 2.2. Protein Production and Purification

After 72 h induction, culture supernatants were collected by centrifugation (4000× *g*, 10 min, 4 °C) and adjusted to pH 8.0. Purification was performed by nickel-nitrilotriacetic acid (Ni^2+^-NTA) IMAC using standard binding (20 mM sodium phosphate, 300 mM NaCl, pH 8.0), wash (20 mM imidazole), and elution (250 mM imidazole) buffers [12].

The eluate was sequentially processed using 30 kDa and 3 kDa molecular weight cut-off (MWCO) ultrafiltration cassettes to remove high-molecular-weight contaminants, concentrate the protein, and exchange the buffer (20 mM sodium acetate, pH 5.4, 0.01% Tween-20 (Merck, NJ, USA)).

Protein purity and identity were confirmed by 16.5% SDS-PAGE with silver staining and His-tag-specific in-gel fluorescence (InVision™, Invitrogen, Waltham, MA, USA). Protein concentration was determined by BCA assay.

### 2.3. Structural Characterization and In Silico Analysis

The identity and molecular mass of the purified peptide were confirmed by LC–MS/MS following tryptic digestion and peptide mass fingerprint analysis against the theoretical BbroAFP sequence.

Structural and physicochemical properties were evaluated using bioinformatic approaches. The monomeric structure of BbroAFP was predicted using AlphaFold 3 [13], and model quality was assessed based on predicted Local Distance Difference Test (pLDDT) scores. Structural features such as cysteine distribution, disulfide bond formation, and overall fold compactness were analyzed.

To assess potential oligomerization, homodimeric structure prediction was performed using AlphaFold2-Multimer via ColabFold v1.5.5 [14]. Two identical sequences were modeled as separate chains under default multimer settings. Model quality was evaluated using predicted TM-score (pTM), inter-chain TM-score (ipTM), and Predicted Alignment Error (PAE). Only high-confidence models (pLDDT > 90, ipTM > 0.80, low inter-chain PAE) were selected for further analysis.

The top-ranked model was analyzed in UCSF ChimeraX (v1.x) to calculate buried surface area (BSA) and solvent-accessible surface area (SASA). ΔSASA values were used to estimate interface size and surface reduction, with calculation details provided in Table S4. Interface residues were defined based on non-zero buried surface area contributions. Electrostatic and hydrophobic surface properties were visualized using ChimeraX tools [15]. Binding affinity was estimated using the PRODIGY web server at 25 °C, providing predicted binding free energy (ΔG), dissociation constant (Kd), and interface interaction parameters [16].

### 2.4. Antimicrobial Activity Assay

The minimum inhibitory concentration (MIC) of BbroAFP against *B. cinerea* was determined using a broth microdilution method adapted from CLSI M38-A2 with slight modifications [17]. Conidia from 7 to 10-day-old malt extract agar (MEA) cultures were suspended in 0.2 × malt extract broth (MEB) and adjusted to 2 × 10^4^ conidia/mL. Equal volumes of conidia suspension and peptide solution were mixed in 96-well plates (final conidia concentration: 1 × 10^4^ conidia/mL; BbroAFP: 32–0.25 μM) and incubated at 21 °C in the dark. OD_600_ was measured at 0, 24, 48, and 72 h. MIC values against other filamentous fungi (*Verticillium dahliae*, *Alternaria alternata*, *Fusarium oxysporum*, *Penicillium expansum*, *Aspergillus niger*, and *Aspergillus flavus*) were determined using the same protocol.

Antifungal activity against *Candida albicans* was assessed using CLSI M27-A3 [18]. Yeast suspensions (1 × 10^5^ CFU/mL) in 0.2× MEB were mixed with serially diluted BbroAFP in 96-well plates and incubated at 28 ± 1 °C. OD_600_ was recorded at 0, 24, and 48 h. Antibacterial activity against *Escherichia coli* and *Staphylococcus aureus* was evaluated using CLSI M07-A9 [19]. Bacterial suspensions (1 × 10^6^ CFU/mL) in Mueller–Hinton broth were incubated with BbroAFP in 96-well plates at 36 ± 1 °C, and OD_600_ was measured at 0 and 24 h. Amphotericin B (1 μg/mL) and Kanamycin A (50 μg/mL) were used as positive controls for fungal and bacterial assays, respectively. MIC was defined as the lowest concentration completely inhibiting growth. Data are presented as mean ± SD. All assays were performed in triplicate.

### 2.5. Temperature and pH Stability Assay

BbroAFP stability was evaluated under different pH and temperature conditions [20].

For pH stability, the peptide was incubated at pH 3, 7.4, and 9.5 for 1 h, followed by buffer exchange to pH 5.4 and MIC testing.

For thermal stability, samples were incubated at 25, 50, and 100 °C for 1 h prior to MIC analysis. Residual activity (1 μM peptide) was quantified by OD_600_ at 72 h and expressed relative to untreated controls (pH 5.4, 25 °C). Data are presented as mean ± SD. All assays were performed in triplicate.

### 2.6. Fluorescence Labeling and Confocal Microscopy

#### 2.6.1. TUNEL Assay

Apoptosis-like DNA fragmentation was assessed using the terminal deoxynucleotidyl transferase dUTP nick end labeling (TUNEL) assay. Hyphae of *B. cinerea* (12–16 h old) treated with BbroAFP for 3 h were fixed in 4% paraformaldehyde, permeabilized with 0.1% Triton X-100, and labeled using an in situ cell death detection kit (Invitrogen). Nuclei were counterstained with 4′,6-diamidino-2-phenylindole (DAPI) to check for co-localization of the TUNEL signal [21].

#### 2.6.2. Internalization Study

To track the spatiotemporal dynamics of peptide interaction with fungal cells, BbroAFP was fluorescently labeled using BODIPY-FL-EDA (Invitrogen). The fluorophore was conjugated to the carboxyl groups of the proteins using 1-ethyl-3-(3-dimethylaminopropyl)carbodiimide (EDC)/N-hydroxysuccinimide (NHS) coupling chemistry, followed by dialysis to remove unreacted dye [22]. To confirm that the covalent attachment of the fluorophore did not alter the peptide’s biological function, preliminary MIC assays were conducted comparing labeled and unlabeled BbroAFP; no significant differences in antifungal potency were observed.

Sample Preparation: *B. cinerea* conidia were germinated in 10% MEB on sterile glass-bottom 35 mm dishes in the dark at 21 °C for 12–16 h until germ tubes were established.

Imaging and time-lapse acquisition (Video S1): Labeled BbroAFP (1 μM, MIC) was added to germinated *B. cinerea* hyphae, and time-lapse imaging was performed at ~8–10 s intervals to monitor peptide internalization. Data acquisition and processing were carried out using Zeiss ZEN 3.3 (blue edition). Fluorescent markers included BODIPY for peptide localization (Ex 488 nm/Em 520 nm), Calcofluor White for cell wall staining (CFW; Ex 405 nm/Em 410 nm), propidium iodide for membrane integrity (PI; Ex 555 nm/Em 575 nm), and DAPI for nuclear visualization (Ex 405 nm/Em 410 nm). Imaging was performed on a LSM 700 confocal microscope using a Plan-Apochromat 63×/1.40 oil immersion objective (Zeiss, Oberkochen, Germany). Images were acquired at 512 × 512 resolution (113 μm field of view; pixel dwell time 3.15 μs) in three-track 12-bit mode with excitation at 405, 488, and 555 nm. The pinhole was set to 1 Airy unit, and emission signals were detected using a PMT system (Zeiss, Oberkochen, Germany) with appropriate dichroics and emission filters. A transmitted light channel was recorded in parallel. Detector gain values were optimized for each channel. Subsequent image processing, including brightness and contrast adjustments, was carried out using ImageJ v1.54k (NIH, Bethesda, MD, USA).

### 2.7. ROS Determination

Intracellular ROS levels were quantified using the oxidation-sensitive probe 5-(and-6)-chloromethyl-2′,7′-dichlorodihydrofluorescein diacetate (CM-H_2_DCFDA) (10 μM, Invitrogen). *B. cinerea* hyphae (12–16 h old) were loaded with the probe and treated with BbroAFP (0.25–4 μM). Fluorescence intensity was monitored kinetically every minute for 15 min using a fluorescence microplate reader [23]. To establish a comparative baseline for ROS induction, hydrogen peroxide (H_2_O_2_, 1–5 mM) was included as a positive control. All kinetic fluorescence data were normalized to untreated control levels to account for background auto-fluorescence. Each assay was performed in triplicate (*n* = 3), and the results are presented as mean ± standard error of the mean (SEM). For microplate measurements, the excitation and emission wavelengths were set at 485 nm and 530 nm, respectively.

### 2.8. Gene Expression Analysis

To investigate transcriptional responses associated with BbroAFP-induced cell death, expression levels of selected apoptosis-related genes were analyzed.

Total RNA was extracted from 24 h-old *B. cinerea* mycelia (50 mg) following 0.5 and 3 h treatments with BbroAFP (2, 1, and 0.5 × MIC). RNA isolation was performed using the Plant/Fungi Total RNA Isolation Kit (Norgen, Thorold, ON, Canada) with on-column DNase I digestion. RNA quality and concentration were assessed using a NanoDrop One spectrophotometer (Thermofischer Scientific, Waltham, MA, USA) (A260/A280 > 2.0).

qRT-PCR was conducted using Luna One-Step Master Mix (NEB, Ipswich, MA, USA) on a Bio-Rad CFX96 system (Bio-Rad Laboratories, Hercules, CA, USA). Each 20 μL reaction contained 10 μL master mix, 0.4 μL of each primer (10 μM), 2 μL RNA template, and nuclease-free water. The following genes were analyzed: *bcaif1* (apoptosis-inducing factor), *bcmca1* and *bcmca2* (metacaspases), and *bctubA* (reference gene). Primer details are provided in Table 1. The stability of *bctubA* was supported by previous reports and experimentally confirmed in this study (Ct 17.50–17.83; SD < 0.6) [24]. Each of the three biological replicates included 16 technical replicates. Thermal cycling conditions consisted of 95 °C for 1 min followed by 40 cycles of 95 °C for 10 s and 60 °C for 30 s. Relative expression was calculated using the 2^−ΔΔCt^ method with untreated samples as a calibrator. Statistical analyses were performed on ΔCt values using two-way analysis of variance (ANOVA) with Dunnett’s test. Primer specificity was confirmed by melt curve analysis (60–95 °C), and reproducibility (>99%) was validated across independent biological replicates (Table S5).

### 2.9. Dose–Response and Cytotoxicity Assessment of BbroAFP in Caco-2 and HepG2 Cell Lines

Caco-2 and HepG2 cells were cultured under standard conditions (37 °C, 5% CO_2_) in EMEM supplemented with 10% FBS and 1% penicillin/streptomycin. Cells were seeded in 96-well plates (5 × 10^3^ cells/well) and allowed to attach for 24 h.

For dose–response analysis, cells were treated with BbroAFP (0.19–100 μM) prepared in sodium acetate buffer. Vehicle and untreated controls were included, with buffer concentrations kept constant across all conditions. After 24 h, cell viability was assessed using the cell viability tetrazolium salt (WST-8) assay (Sigma-Aldrich, St. Louis, MO, USA) following the manufacturer’s instructions. Briefly, 10 μL of reagent was added per well, incubated for 2 h at 37 °C, and absorbance was measured at 440 nm. Viability was expressed relative to untreated controls.

Membrane integrity was evaluated using a lactate dehydrogenase (LDH) release assay (NutriCulture, EcoTech Biotechnology, Istanbul, Türkiye). Supernatants were collected post-treatment, and absorbance was measured at 490 nm. Cytotoxicity was calculated relative to total LDH release after lysis correction.

Morphological changes were examined by inverted phase-contrast microscopy (×20), and representative images were recorded. All experiments were performed in triplicate and repeated independently three times. Data are presented as mean ± SD, and statistical significance was determined by one-way ANOVA with Dunnett’s post hoc test.

### 2.10. Detached Leaf Assay

To evaluate the in vivo antifungal efficacy of BbroAFP, a detached leaf assay was performed using tomato (*Solanum lycopersicum*) leaves. Plants were grown under controlled conditions (12 h light/dark, 23/20 °C day/night, 55–60% humidity), and leaves were collected from 4-week-old plants and maintained in humid chambers.

BbroAFP (1 μM) and *B. cinerea* conidia (1 × 10^7^ conidia/mL in 0.1× MEA) were applied under four conditions: (A) infection control, (B) BbroAFP only (phytotoxicity control), (C) BbroAFP + *B. cinerea* (treatment), and (D) uninfected control (0.1× MEB). A 10 μL droplet was applied to two positions on the abaxial leaf surface and allowed to air-dry. Leaves were incubated in sterile Petri dishes with moist filter papers at 21 °C for 5 days (*n* = 6 leaves per group).

Fungal colonization and cell death were assessed using Evans Blue staining [25]. Leaves were stained (0.025% *w*/*v*, 30 min), rinsed, and decolorized in 96% ethanol (15 min), then preserved in glycerol:ethanol:water (4:2:4, *v*/*v*/*v*). Images were captured using a Canon EOS M50 Mark II (Tokyo, Japan) and processed with ImageJ (NIH, USA). Blue staining indicated loss of membrane integrity associated with fungal infection and cell death.

### 2.11. Statistical Analysis

Statistical analyses were performed using GraphPad Prism v10. Dose–response curves were fitted with a four-parameter logistic (4PL) nonlinear regression model to calculate IC_50_ values and 95% confidence intervals (CI). Data from cell viability (WST-8) and cytotoxicity (LDH) assays are expressed as mean ± standard deviation (SD). Transcriptional data (qRT-PCR) and kinetic ROS levels are presented as mean ± standard error of the mean (SEM) derived from at least three independent biological replicates. Statistical significance was evaluated using one-way or two-way ANOVA followed by Dunnett’s or Tukey’s post hoc tests for multiple comparisons. A *p*-value < 0.05 was considered statistically significant (* *p* < 0.05, ** *p* < 0.01, *** *p* < 0.001, **** *p* < 0.0001).

## 3. Results

### 3.1. Recombinant Protein (BbroAFP) Production and Purification

**Figure 1:**
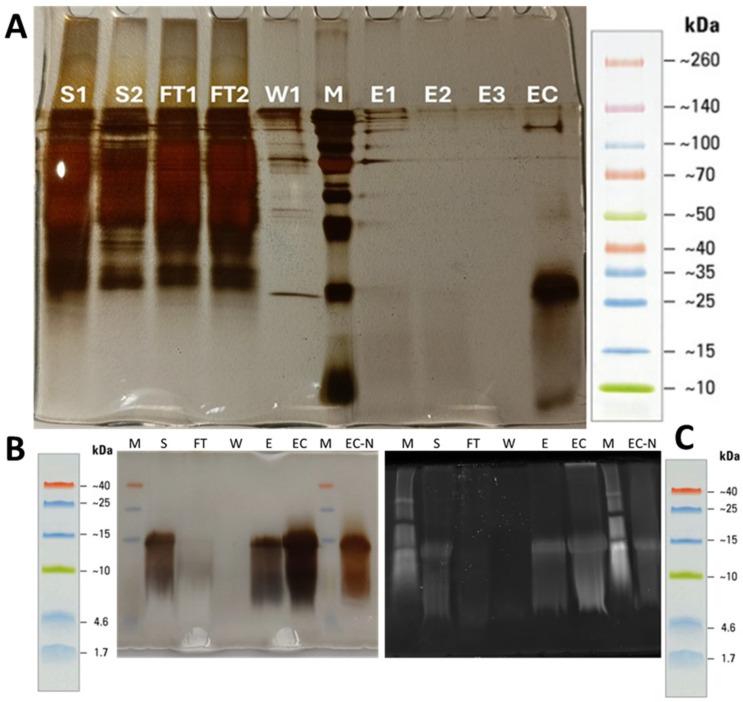
SDS-PAGE analysis of BbroAFP expression and purification. (**A**) Silver-stained gel showing large-scale expression and purification. Lane M: molecular weight marker (10–260 kDa); S1–S2: soluble fractions; FT1–FT2: flow-through; W1: wash; E1–E3: elution fractions; EC: concentrated eluate. (**B**) Silver-stained gel of the final purified fraction. (**C**) His-tag-specific in-gel fluorescence analysis. Lane M: low-range marker (1.7–40 kDa); S: supernatant; FT: flow-through; W: wash; E: elution; EC: concentrated eluate; EC-N: non-denatured concentrated eluate.

The coding sequence of BbroAFP was successfully cloned and heterologously expressed in *P. pastoris* KM71H. Following methanol induction, SDS–PAGE analysis of the culture supernatant revealed a protein band below 10 kDa, consistent with the predicted size of the mature peptide (~9.2 kDa, including the C-terminal His-tag) (Figure 1). Protein expression was monitored at different cell densities, with OD_600_ values of 30 and 100 showing comparable secretion levels (Figure 1A, lanes S1–S2).

A two-step purification strategy efficiently enriched BbroAFP. IMAC selectively captured the His-tagged peptide, followed by sequential ultrafiltration using 30 kDa and 3 kDa MWCO membranes to remove host-derived contaminants, concentrate the protein, and perform buffer exchange. Silver-stained SDS–PAGE of the final fraction showed a predominant low-molecular-weight band corresponding to monomeric BbroAFP (~10–15 kDa) and a higher-molecular-weight species consistent with dimer-like forms, indicating high purity (>95%) (Figure 1). Minimal host protein contamination was observed in concentrated fractions (EC and EC-N), and His-tag-specific fluorescence confirmed the identity of the recombinant peptide. His-tag-specific in-gel fluorescence (Figure 1C) further verified the identity of the monomer (~9.5 kDa) and the presence of dimer-like species.

**Figure 2:**
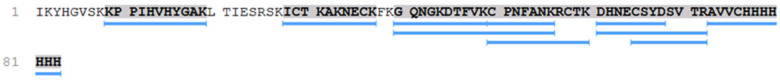
LC-MS/MS peptide mapping of BbroAFP. The amino acid sequence coverage obtained by LC-MS/MS analysis is shown. Blue bars represent detected peptide fragments matching the theoretical sequence of the mature peptide.

LC–MS/MS peptide mapping confirmed protein identity and integrity, with 78% sequence coverage and 10 unique peptides matching the theoretical sequence (Figure 2). A total of 270 peptide spectrum matches (PSMs) were obtained, corresponding to a high identification confidence (−10logP = 143.53). The experimentally determined molecular mass (9.548 kDa) closely matched the predicted value. Protein identification was validated at both peptide and protein levels (FDR < 1%), with no evidence of truncation or degradation.

In silico sequence and phylogenetic analyses indicated that BbroAFP clusters with classical PAF-like cysteine-rich antifungal proteins and retains conserved features, including a signal peptide, pro-peptide region, and cysteine framework (Figure S1).

### 3.2. In Silico Structural Characterization

**Figure 3:**
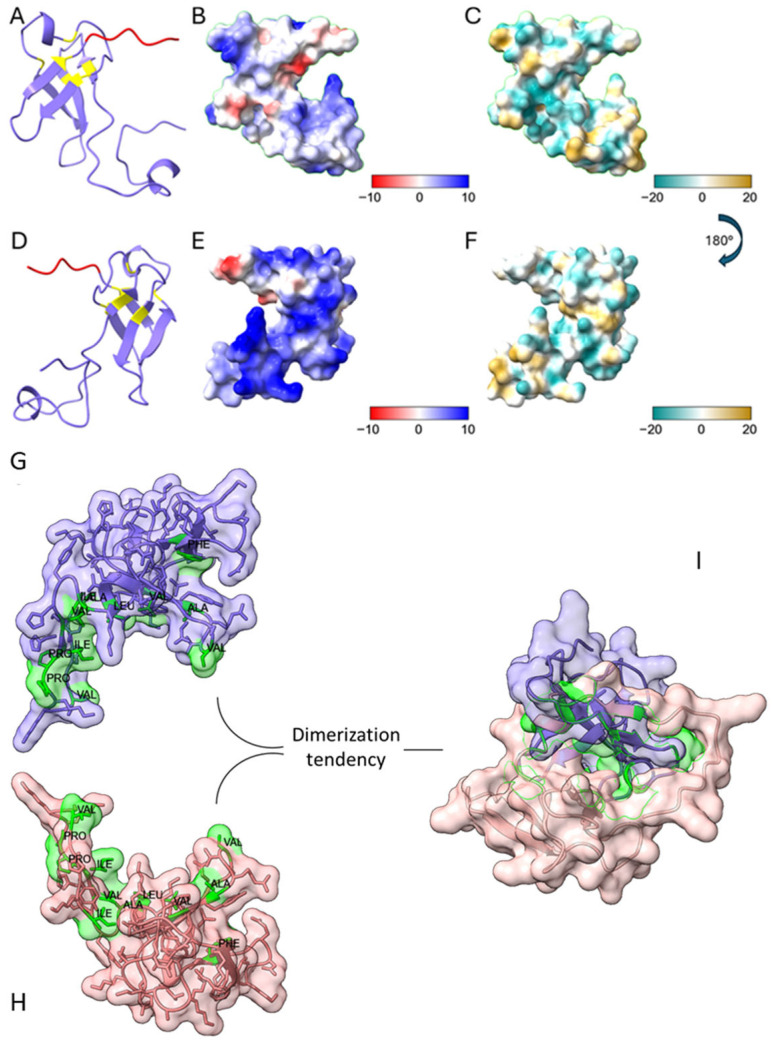
In silico structural characterization of the mature BbroAFP predicted by AlphaFold 3. (**A**,**D**) Ribbon representations showing cysteine residues (yellow) and the C-terminal His-tag (red); six cysteines form three intramolecular disulfide bonds. (**B**,**E**) Electrostatic surface potential maps at pH 7.4 (−10 to +10), with positively charged regions in blue and negatively charged regions in red. (**C**,**F**) Hydrophobicity surface maps (−20 to +20), with hydrophilic regions in cyan and hydrophobic regions in yellow. Panels (**A**–**C**) and (**D**–**F**) represent two orientations of the monomer. (**G**,**H**) Surface-exposed hydrophobic residues (green) highlighting discrete hydrophobic patches. (**I**) Predicted a homodimer model showing a symmetric dimerization interface.

AlphaFold 3 modeling predicted a compact, globular structure for BbroAFP, consistent with cysteine-rich antifungal proteins. The model contained six cysteine residues forming three intramolecular disulfide bonds, supported by sulfur–sulfur distances of ~2 Å, defining a tightly packed and stable core (Figure S2; Figure 3A,D).

Electrostatic surface analysis revealed a polarized topology with cationic regions and hydrophobic patches (Figure 3), consistent with amphipathic, membrane-active proteins and suggesting interactions with negatively charged fungal surfaces.

Homodimer prediction using AlphaFold Multimer [14] showed high confidence (pLDDT > 90, pTM: 0.85–0.90, ipTM: 0.80–0.90), with low interfacial PAE indicating reliable inter-chain interactions. ChimeraX analysis [15] revealed a buried surface area (BSA) of 1703.1 Å^2^, exceeding thresholds for biologically relevant dimers (>1500 Å^2^; Table S1). SASA values were 6727.6 Å^2^ and 6733.6 Å^2^ for chains A and B, respectively, and dimerization resulted in a ΔSASA of 3406.2 Å^2^ (~25.3% burial per monomer), involving 82 interface residues (Table S2).

Key interface residues included LYS19 (~163 Å^2^), TYR16 (~150 Å^2^), VAL70 (~110 Å^2^), ILE12 (~100 Å^2^), TYR3 (~99 Å^2^), and GLU23 (~91 Å^2^) (Table S3). Although hydrophobic residues represented 31.7% of interface residues (26.8% excluding tyrosine), they contributed disproportionately to stabilization, accounting for ~1562 Å^2^ (~91.7%) of total BSA and ~1062 Å^2^ (~62.4%) without tyrosine.

Thermodynamic analysis using PRODIGY [16] predicted a binding free energy (ΔG) of −12.4 kcal·mol^−1^ and a dissociation constant (Kd) of 8.2 × 10^−10^ M, consistent with high-affinity dimerization. A total of 116 intermolecular contacts were identified across different interaction types, with apolar and charged non-interacting surface residues accounting for 32.86% and 31.43%, respectively.

Overall, AlphaFold modeling, ChimeraX analysis, and PRODIGY predictions consistently support a structurally stable and biologically relevant BbroAFP homodimer, characterized by a hydrophobic core within a cationic, amphipathic surface (Figure 3I).

### 3.3. Antimicrobial Spectrum and Stability Profile

BbroAFP exhibited a pronounced and selective antifungal activity profile against a diversity of filamentous fungi and yeast while showing no significant activity against the tested bacterial strains (Table 2). BbroAFP demonstrated potent antifungal activity, with the highest sensitivity observed against *V. dahliae* (MIC < 0.25 µM). Significant inhibitory effects were also recorded for *B. cinerea*, *A. alternata*, *A. niger*, and *P. expansum*, all exhibiting MIC values of 1 µM (Table 2). Moderate antifungal activity was observed against *F. oxysporum*, which was inhibited at MIC values of 2 µM, whereas *C. albicans* displayed reduced susceptibility (MIC = 16 µM; Table 2). In contrast, *A. flavus* showed resistance to BbroAFP even at the highest concentration tested (MIC > 32 µM).

No inhibitory effect was detected against the Gram-negative bacteria *E. coli* or the Gram-positive bacteria *S. aureus* (MIC > 32 µM; Table 2), indicating a strong preference of BBroAFP for fungal targets. This species-specific susceptibility pattern suggests that variations in fungal cell wall composition, membrane lipid organization, or surface charge properties may influence peptide–cell interactions and determine sensitivity to BBroAFP. Control experiments confirmed that BODIPY-labeled BbroAFP retained antifungal activity comparable to the unlabeled peptide, indicating that fluorescent labeling did not significantly affect peptide function. The validity of the assay conditions was confirmed using appropriate positive controls. Amphotericin B, a well-established antifungal agent, was included as a reference control in fungal assays and consistently inhibited fungal growth under the tested conditions. Kanamycin A, a broad-spectrum antibacterial agent, was used as a positive control for bacterial assays and confirmed the responsiveness of the bacterial strains.

### 3.4. Stability Profile of BbroAFP

The stability of BbroAFP was evaluated under various abiotic conditions, including different temperatures and pH values (Table 3). Residual antifungal activity (1 µM) was measured at 72 h following a 1 h pre-incubation under each condition.

BbroAFP exhibited high stability across all tested pH ranges. Under acidic conditions (pH 3.0), 98.4% activity was retained, while activity remained at 94.6% at pH 7.4 and 97% at pH 9.5, indicating strong tolerance to pH variation (Table 3).

Thermal stability analysis showed that activity was largely preserved at moderate temperatures, with 96.3% and 96.9% activity retained after incubation at 25 °C and 50 °C, respectively. Although exposure to 100 °C for 1 h reduced activity to 60.6%, the peptide remained partially active (Table 3).

Overall, these results demonstrate that BbroAFP possesses a structurally robust fold resistant to both pH and thermal stress, consistent with cysteine-rich antifungal proteins stabilized by disulfide bonds. All values are presented as mean ± SD from three independent biological replicates with technical triplicates. Percentage inhibition was calculated relative to untreated controls, with low variability indicating high reproducibility under all tested conditions.

### 3.5. Spatiotemporal Dynamics of BbroAFP Internalization and Antifungal Action

**Figure 4:**
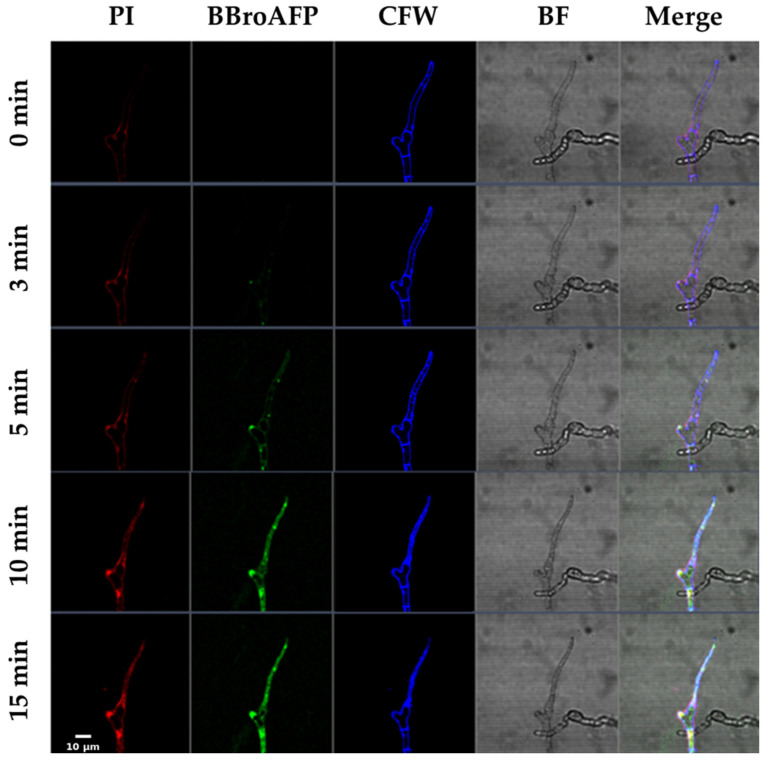
Spatiotemporal dynamics of BbroAFP internalization and membrane permeabilization in *B. cinerea* hyphae. Time-lapse confocal laser scanning microscopy of *B. cinerea* hyphae treated with BODIPY-labeled BbroAFP (green). Propidium iodide (PI-red) was used to monitor membrane integrity and Calcofluor White (CFW, Blue) to stain cell walls. Scale bar: 10 µm.

**Figure 5:**
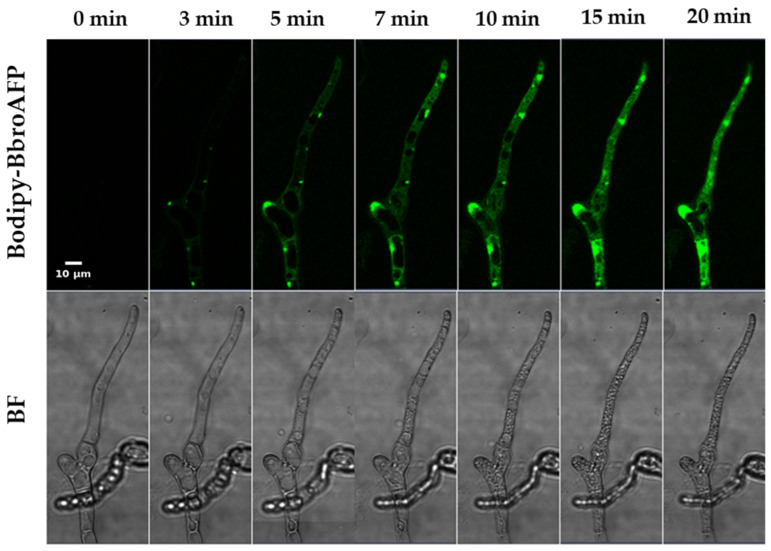
Rapid cytosolic accumulation of BbroAFP and vacuolar exclusion precede hyphal collapse. Spatiotemporal accumulation of BODIPY-labeled BbroAFP (green) in *B. cinerea* hyphae. Corresponding bright-field (BF) images illustrate morphological changes during the treatment period. Scale bar: 10 µm.

Time-lapse confocal microscopy was employed to investigate the spatiotemporal interaction between BbroAFP and *B. cinerea* hyphae (Figure 4 and Figure 5).

0–3 min (Binding Phase). BODIPY-labeled BbroAFP rapidly associated with the fungal cell surface, with preferential accumulation at hyphal tips and septal junctions—regions associated with active cell wall remodeling and polarized growth. During this early phase, the membrane-impermeant dye propidium iodide (PI) remained excluded from the intracellular space, indicating that plasma membrane integrity was preserved.

5–10 min (Internalization Phase). Following surface accumulation, BbroAFP-associated fluorescence progressively appeared and accumulated within the intracellular region of the hyphae. This redistribution of fluorescence coincided with the onset of PI uptake, suggesting peptide-associated membrane permeabilization.

10–15 min (Terminal Phase). At later time points, fluorescence signals became broadly distributed within the intracellular space of the hyphae but appeared largely excluded from large vacuole-like compartments, resulting in a characteristic “vacuolar exclusion” pattern. Concomitantly, strong intracellular PI staining was observed, consistent with loss of membrane integrity and cell death. Bright-field imaging revealed intracellular structural alterations, including the appearance and enlargement of vacuole-like compartments, followed by loss of turgor pressure and collapse of cytoplasmic organization, further supporting the terminal antifungal effect of BbroAFP (Figure 5). Notably, Calcofluor White staining demonstrated that the overall cell wall architecture remained preserved throughout the process, indicating that BbroAFP primarily affects cellular membranes rather than inducing gross cell wall disruption.

### 3.6. Mechanism of Action: ROS and Regulated Cell Death-like Processes

**Figure 6:**
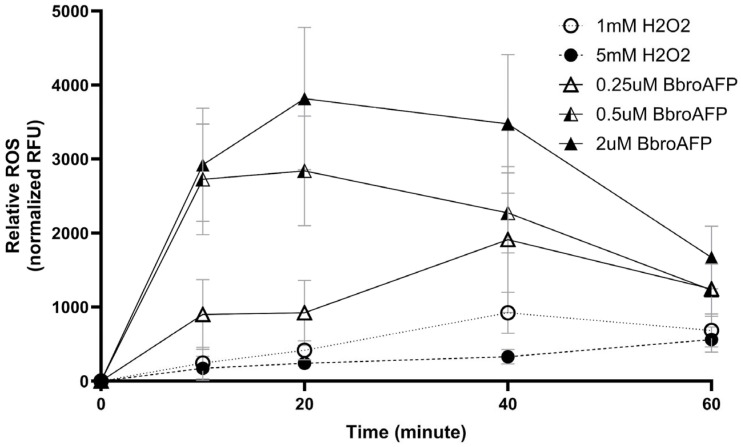
Kinetic monitoring of intracellular ROS levels in *B. cinerea* following treatment with BbroAFP (0.25–2 µM) and H_2_O_2_ controls (1–5 mM). Data are normalized to untreated controls and presented as mean ± SEM (*n* = 3).

Intracellular ROS production was monitored using CM-H_2_DCFDA following BbroAFP treatment (0.25, 0.5, and 2 µM) and compared with H_2_O_2_-induced oxidative stress (1 and 5 mM) (Figure 6).

BbroAFP induced a rapid, dose-dependent ROS increase. At 0.5 and 2 µM, ROS levels peaked within 10–20 min, whereas at 0.25 µM, the peak occurred at ~40 min. Higher concentrations accelerated ROS onset, with 2 µM producing the strongest response. ROS levels subsequently declined to near baseline by 60 min, indicating a transient oxidative burst.

In contrast, H_2_O_2_ induced a slower and lower-amplitude ROS increase over the same period (Figure 6). Extended time-course analysis confirmed that BbroAFP triggers a rapid and transient ROS response, whereas H_2_O_2_ produces a slower, more sustained effect (Figure S3). Data are presented as mean ± SEM (*n* = 3) and were reproducible across experiments. A schematic summary is provided in Figure S4.

• Nuclear Fragmentation and DNA Damage

**Figure 7:**
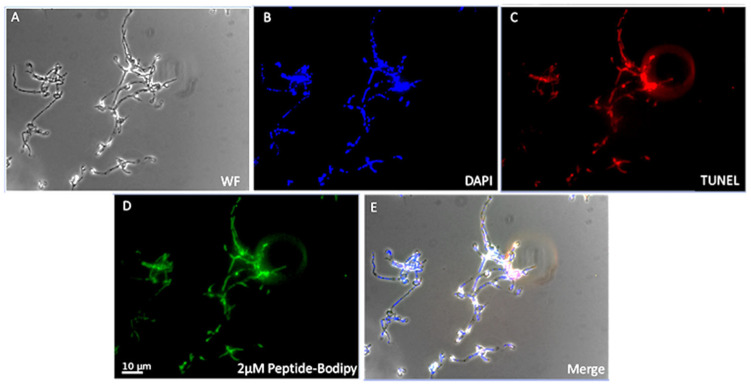
Fluorescence microscopy analysis of BbroAFP localization and its association with fungal cell death. Representative fluorescence microscopy images illustrate the intracellular localization of BODIPY FL–conjugated BbroAFP and nuclear DNA fragmentation in fungal hyphae. (**A**) Bright-field (BF) image showing overall hyphal morphology. (**B**) DAPI staining (blue) highlights fungal nuclei. (**C**) TUNEL assay (Alexa Fluor 647, red) indicating nuclear DNA fragmentation. (**D**) BODIPY FL–labeled BbroAFP (green) showing intracellular peptide localization. (**E**) Merged image. Scale bar: 10 µm. See also Figure S5 for additional data.

DNA fragmentation was assessed using the TUNEL assay. BbroAFP-treated hyphae exhibited strong TUNEL-positive signals co-localizing with DAPI-stained nuclei, whereas untreated controls showed no signal (Figure 7). DNase I-treated samples confirmed assay specificity, and no basal DNA fragmentation was detected in controls (Figure S5).

Although TUNEL positivity indicates DNA fragmentation, it is not exclusive to apoptosis. Therefore, these results are interpreted as evidence of peptide-associated nuclear damage rather than definitive apoptosis.

Collectively, BbroAFP treatment is associated with rapid ROS accumulation followed by nuclear DNA damage. Together with peptide internalization, these findings support activation of a regulated cell death–like response rather than nonspecific cytotoxicity.

### 3.7. Gene Expression Analysis of Stress-Related Cell Death Markers in B. cinerea

**Figure 8:**
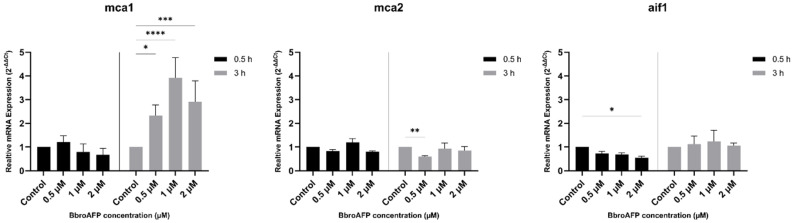
Relative fold-change in the expression (2^−ΔΔCt^) of pro-apoptotic genes (*bcmca1*, *bcmca2*, and *bcaif1*) in *B. cinerea* hyphae following treatment with BbroAFP (0.5, 1, and 2 µM) for 0.5 h and 3 h. Expression levels were normalized to the reference gene *bctubA* and are presented as fold-change relative to untreated controls. Data represent the mean ± SEM of three independent biological replicates. Asterisks indicate statistical significance compared to untreated controls (* *p* < 0.05; ** *p <* 0.01; *** *p* < 0.001; **** *p* < 0.0001).

To assess cellular responses to BbroAFP, the expression of metacaspase genes (*mca1* and *mca2*) and the apoptosis-inducing factor homolog (*aif1*) were analyzed by qRT-PCR at 0.5 h and 3 h following peptide treatment (Figure 8). Expression levels were evaluated at 0.5, 1, and 2 μM relative to untreated controls. Primer specificity and technical reproducibility were confirmed by melt curve analysis (Table S5).

*mca1* expression remained close to control levels at 0.5 h across all concentrations, suggesting no pronounced early transcriptional response. At 3 h, *mca1* expression increased relative to control, particularly at higher peptide concentrations, indicating a delayed transcriptional response.

*mca2* expression was largely stable under most conditions. At 0.5 h, transcript levels were comparable to control, while at 3 h, a slight decrease was observed at 0.5 μM, with no consistent changes at higher concentrations.

In contrast, *aif1* showed minor fluctuations, with a modest decrease at 0.5 h at specific concentrations, but no consistent or statistically significant changes at 3 h across treatments.

Overall, BbroAFP induced moderate and time-dependent transcriptional changes. The observed increase in *mca1* expression at later time points, together with largely stable *mca2* and *aif1* expression, suggests that peptide-induced stress does not lead to a clear or sustained activation of canonical metacaspase-dependent pathways under the tested conditions.

### 3.8. In Vitro Safety Assessment in Human Cell Lines

**Figure 9:**
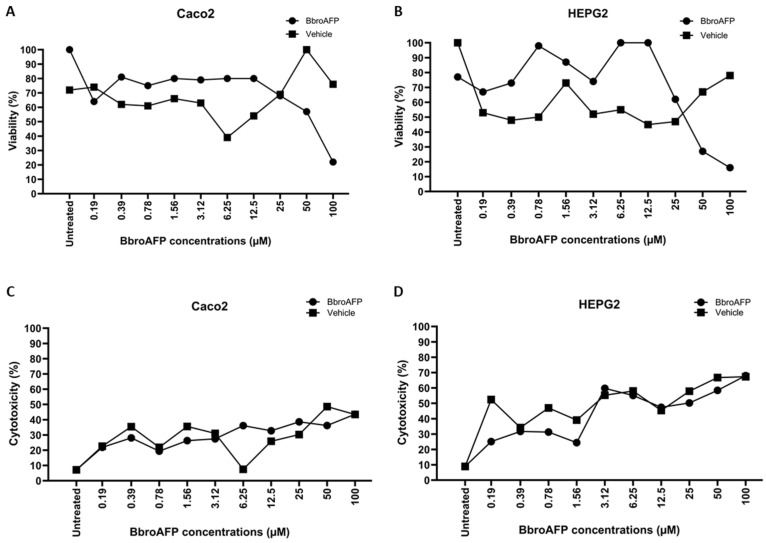
Cytotoxicity profile of BbroAFP on human Caco-2 and HepG2 cell lines. (**A**,**B**) Cell viability and (**C**,**D**) LDH release in Caco2 (**A**,**C**) and HepG2 (**B**,**D**) cells treated with BbroAFP (0.19–100 μM). (•) represents cells treated with BbroAFP, while (▪) indicates the vehicle control (sodium acetate buffer). Untreated cells served as negative control. The data represent mean ± SD from three independent experiments.

To evaluate the safety profile of BbroAFP, its cytotoxicity was assessed following 24 h of exposure to increasing peptide concentrations (0.19–100 μM) in human Caco-2 and HepG2 cells using WST-8 metabolic activity and LDH membrane integrity assays. In both assays, data were normalized to untreated negative controls to calculate percentage viability and cytotoxicity, respectively. In Caco-2 cells, BbroAFP induced a concentration-dependent decrease in cell viability. However, no significant reduction in viability was observed at low-to-moderate concentrations (≤12.5 μM). A significant decline in viability was only evident at concentrations ≥25 μM. The calculated IC_50_ value for Caco-2 cells was 72.5 μM (95% CI: 39.7–92.4 μM). In contrast, the vehicle control (sodium acetate buffer) showed a non-progressive response without a determinable IC_50_, indicating that the BbroAFP peptide itself, rather than the buffer, is responsible for the observed effects at high doses. LDH assays corroborated these findings, showing low cytotoxicity (<30%) at ≤12.5 μM, which increased to approximately 36% at 100 μM. Elevated LDH release was also detected in vehicle-treated samples at higher concentrations, suggesting that the mild membrane effects observed may be partially attributable to the buffer composition (Figure 9A,C). Phase-contrast microscopy revealed dose-dependent morphological alterations consistent with the quantitative viability and LDH data (Figure S6).

HepG2 cells exhibited a similar tolerance profile at lower concentrations. Viability remained comparable to untreated controls in the range of 0.19–12.5 μM. At higher concentrations (≥25 μM), a progressive, dose-dependent reduction in viability was observed, resulting in an IC_50_ of 27.8 μM (95% CI upper bound: 45.0 μM). The LDH release assay indicated elevated cytotoxicity (>50%) at concentrations ≥3.12 μM; however, the vehicle control induced similarly high levels of LDH release (Figure 9B,D). This suggests that HepG2 cells are particularly sensitive to the sodium acetate buffer, and the observed membrane compromise is likely an artifact of the vehicle rather than specific toxicity of the BbroAFP peptide. Morphological examination by phase-contrast microscopy showed dose-dependent changes, including increased cell rounding and reduced monolayer density at higher concentrations, consistent with the quantitative assay results (Figure S7). All results are expressed as the mean ± SD of technical triplicates from three independent biological experiments.

### 3.9. Detached Leaf Assay

*B. cinerea* is a well-characterized necrotrophic fungal pathogen of tomato leaf tissue, causing host cell death during successful infection. Based on the pronounced antifungal activity observed in vitro, the protective potential of the peptide was further evaluated using a detached tomato leaf infection model. Host cell viability was assessed using Evans Blue staining, a well-established method for detecting membrane damage and cell death in plant tissues and previously applied in studies of antifungal proteins [25].

**Figure 10:**
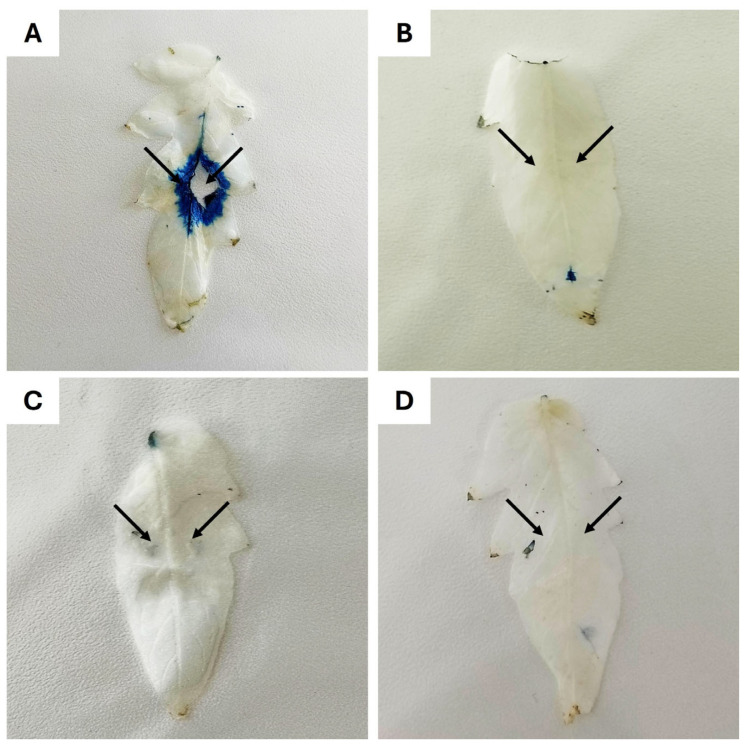
Evans Blue staining of detached tomato leaves to evaluate *B. cinerea* infection and tissue viability. Panels represent the following treatments: (**A**) infection control: *B. cinerea* conidial suspension (1 × 10^7^ conidia/mL), (**B**) BbroAFP phytotoxicity testing: 1 µM BbroAFP, (**C**) plant protection investigation: *B. cinerea* conidial suspension (1 × 10^7^ conidia/mL) with 1 µM BbroAFP, and (**D**) uninfected control: 0.1 × MEB. Arrows indicate the focal application sites where the spore suspension and BbroAFP were co-applied and analyzed at 5 days post-inoculation (dpi). Arrows indicate inoculation areas.

To evaluate potential phytotoxic effects, uninfected leaves were treated with the BbroAFP peptide alone. These leaves did not exhibit Evans Blue staining, necrotic lesions, or visible tissue damage, indicating that the BbroAFP peptide is non-phytotoxic at the applied concentration (Figure 10D). Similarly, buffer-treated control leaves remained unstained and healthy throughout the incubation period (Figure 10C). In contrast, leaves inoculated with *B. cinerea* alone developed extensive necrotic lesions accompanied by intense Evans Blue staining at and around the inoculation sites, reflecting widespread host cell death and successful fungal establishment (Figure 10B). When tomato leaves were co-treated with the BbroAFP peptide and *B. cinerea* spores, Evans Blue–positive areas were markedly reduced or entirely absent at the inoculation sites (Figure 10A). The limited staining and absence of pronounced necrotic lesions indicate that the peptide effectively protected tomato leaf tissue against *B. cinerea*–induced necrosis and restricted fungal invasion into host cells. These protective effects were consistently observed across six independent biological replicates.

Evans Blue selectively stains cells with compromised plasma membrane integrity, indicating host cell death at the treatment sites. Extensive blue-stained zones and necrotic lesions were observed in fungus-infected leaves, whereas co-treatment with the peptide markedly reduced Evans Blue staining. No staining or visible tissue damage was detected in buffer-treated or peptide-only leaves, demonstrating the absence of phytotoxic effects. Chlorophyll was removed using ethanol to enhance visualization of necrotic regions. Images are representative of six independent biological replicates.

## 4. Discussion

Cysteine-rich antifungal proteins (AFPs) from filamentous fungi represent a conserved class of defense molecules characterized by high structural stability and selective antifungal activity. Well-studied AFPs such as AFP from *Aspergillus giganteus*, PAF/PAFB from *Penicillium chrysogenum*, NFAP from *Neosartorya fischeri*, and AfpB from *Penicillium digitatum* provide important reference systems for antifungal protein biology [26,27,28,29,30]. In this study, we identified and characterized BbroAFP, a previously undescribed antifungal protein from *B. brongniartii*, and evaluated its antifungal activity against *B. cinerea*. BbroAFP exhibited broad yet selective antifungal activity against filamentous fungi and yeast, with no detectable effect on bacteria. Several phytopathogens were inhibited at low micromolar concentrations, while susceptibility varied across species. Notably, *V. dahliae* showed exceptionally high sensitivity (MIC < 0.25 μM), highlighting strong antifungal potency against this economically important phytopathogen.

Fluorescence microscopy indicated that BbroAFP first associates with the fungal cell surface and subsequently accumulates intracellularly. This sequential localization pattern is consistent with other fungal AFPs and supports a mechanism in which initial interaction with the cell envelope precedes intracellular activity [31].

BbroAFP exposure induced intracellular ROS accumulation, with higher concentrations triggering earlier oxidative responses. ROS generation is a common feature of antifungal protein activity and is frequently associated with mitochondrial dysfunction and stress signaling in filamentous fungi. However, whether ROS directly contributes to cell death in this system remains to be determined [7,31].

DNA fragmentation detected by TUNEL further indicated nuclear damage; however, as TUNEL positivity may result from multiple forms of fungal cell death, these findings should be interpreted alongside ROS accumulation and peptide internalization rather than as definitive evidence of apoptosis. Collectively, the data suggest that BbroAFP triggers oxidative stress–associated cellular responses that may contribute to a non-canonical cell death process, although the precise mechanisms remain to be elucidated.

Gene expression analysis revealed moderate and time-dependent transcriptional responses. *mca1* expression increased at later time points, particularly at higher peptide concentrations, indicating a delayed transcriptional response. In contrast, *mca2* expression remained largely stable across conditions, with minor fluctuations at specific concentrations. *aif1* expression exhibited only modest changes, without a consistent time-dependent pattern.

Overall, these results indicate that BbroAFP induces moderate transcriptional modulation of stress-related genes but does not lead to a clear or sustained activation of canonical metacaspase-associated cell death pathways under the tested conditions.

Structural modeling provided preliminary insights into the dimeric architecture of BbroAFP, which should be ideally coupled with experimental validation. Future studies using NMR, cryo-EM, or SEC–MALS will be necessary to confirm structural features and oligomerization behavior. It should also be noted that the recombinant protein contains a C-terminal His-tag. While such tags are generally minimally disruptive, they may influence small cationic proteins; thus, comparison with tag-free variants would further validate biological activity [32,33].

Detached leaf assays showed no visible phytotoxicity under the test conditions, indicating compatibility with plant tissues. However, further evaluation in whole-plant systems under greenhouse conditions is required.

In addition to its antifungal efficacy, BbroAFP exhibited a favorable in vitro safety profile in human cell lines. No significant reduction in viability was observed in either Caco-2 or HepG2 cells at concentrations up to 12.5 μM, which is substantially higher than its antifungal MIC (1 μM), indicating a safety margin of at least ~12-fold. Cytotoxic effects became apparent only at higher concentrations (≥25 μM), with IC50 values of 72.5 μM in Caco-2 cells and 27.8 μM in HepG2 cells. Notably, LDH assays suggested that part of the observed membrane damage, particularly in HepG2 cells, was attributable to the sodium acetate buffer, indicating that the peptide itself may be less cytotoxic than initially inferred.

These findings are consistent with previous studies on fungal antifungal proteins. For example, the antifungal protein AFP from *A. giganteus* showed no cytotoxic effects on human endothelial cells even at concentrations up to 100 μg/mL and did not induce pro-inflammatory cytokine production (IL-6 and IL-8) [34]. Similarly, AfpB from *P. digitatum* has been reported to induce regulated cell death in fungal cells without causing comparable damage to mammalian systems [27]. Together, these results support that BbroAFP exhibits selective antifungal activity within its effective concentration range and highlight its potential suitability for applications requiring minimal off-target toxicity.

Overall, BbroAFP appears to act through a multi-step mechanism involving cell surface interaction, intracellular accumulation, ROS induction, and stress-associated cellular responses. These findings expand the diversity of AFPs derived from entomopathogenic fungi and highlight BbroAFP as a promising candidate for further antifungal and crop protection studies.

## 5. Conclusions

BbroAFP exhibited potent and selective antifungal activity at low micromolar concentrations against major phytopathogenic fungi while remaining well tolerated by human cells at higher concentrations, indicating a favorable safety margin. The protein also demonstrated high physicochemical stability and showed no phytotoxic effects in detached tomato leaf assays, where it effectively suppressed *B. cinerea*–induced tissue necrosis. Collectively, these results identify BbroAFP as a promising fungal-derived antifungal protein and support its further evaluation as a potential biofungicide. Future studies will focus on whole-plant validation and greenhouse or field applications.

## 6. Patents

A patent application related to the antifungal peptide and its applications described in this study has been filed under the Patent Cooperation Treaty (PCT/TR2026/050149).

## Figures and Tables

**Table 1:** List of specific primers used for genomic verification and qRT-PCR analysis.

Gene Name	Primer Sequence (5′→3′)	Tm (°C)	Amplicon Size (bp)
*bctubA*	F: CAGTTCACGGCTATGTTTCC R: GGGTAGGTTCCAAGTATGAT	59	120
*bcaif1*	F: ATTGCTGGAGCTGGTTTTGC R: TTCCTTGACGGCGTCTATCC	60	93
*bcmca1*	F: GCTTCATCGACAGCCAACTC R: CCAACTTGGCGGAGTAGTTC	60	128
*bcmca2*	F: TACGGCAGTGACAACATCGC R: CGTCGATCTTCACCTTGAGC	59	95

**Table 2:** MIC values of recombinant BBroAFP against filamentous fungi, yeast, and bacteria.

Microorganism	Microbial Group	MIC (μM)
*Botrytis cinerea*	Phytopathogen	1
*Alternaria alternata*	Phytopathogen	1
*Fusarium oxysporum*	Phytopathogen	2
*Penicillium expansum*	Phytopathogen	1
*Aspergillus niger*	Phytopathogen	1
*Aspergillus flavus*	Phytopathogen	>32
*Verticillium dahliae*	Phytopathogen	<0.25
*Candida albicans*	Human Pathogen (Yeast)	16
*Escherichia coli*	Gram-negative Bacteria	>32
*Staphylococcus aureus*	Gram-positive Bacteria	>32

**Table 3:** Antifungal stability of recombinant BbroAFP under various pH and temperature conditions.

Condition (1 h)	% Inhibition (Mean ± SD)
pH 3	98.4 ± 1.3
pH 7.4	94.6 ± 1.8
pH 9.5	97.0 ± 2.5
25 °C	96.3 ± 3.2
50 °C	96.9 ± 1.1
100 °C	60.6 ± 0.3

## Data Availability

The data presented in this study are available on reasonable request from the corresponding author. Public availability is currently restricted due to an ongoing international (PCT) patent application related to the antifungal peptide.

## Supplementary Materials

The following supporting information can be downloaded at https://www.mdpi.com/article/10.3390/jof12050305/s1. Figure S1. Phylogenetic and sequence analysis of BbroAFP and related cysteine-rich antifungal proteins. Figure S2. Structural model of the peptide highlighting cysteine interactions. Figure S3. Extended time-course analysis of intracellular ROS induction following BbroAFP treatment. Figure S4. The schematic illustrates a time-resolved mode of action in which the antifungal peptide initially binds to the fungal cell surface and is rapidly internalized without immediate membrane disruption. Figure S5. Additional TUNEL assay results supporting peptide-induced nuclear DNA fragmentation. Figure S6. Phase-contrast microscopy-based morphological evaluation of Caco-2 cells following 24 h exposure to ten different concentrations of the BbroAFP protein. Figure S7. Phase-contrast microscopy-based morphological evaluation of HepG2 cells following 24 h exposure to ten different concentrations of the BbroAFP protein. Table S1. Global Interface Geometry of the Predicted BbroAFP Homodimer. Table S2. SASA and ΔSASA Comparison Between Monomer and Dimer States. Table S3. Major Interface Residues Ranked by Buried Area. Table S4. Equations of the dimerization analysis in ChimeraX. Table S5. The technical validity of the qPCR assays was confirmed via melt curve analysis across three biological replicates. Video S1. Time-lapse imaging of BbroAFP-induced antifungal action in *B. cinerea* hyphae. References [13,15,35] are cited in the Supplementary Materials.

## Abbreviations

The following abbreviations are used in this manuscript:

4PL	Four-parameter logistic equation
AFP	Antifungal protein
*aif1*	Apoptosis-inducing factor 1
ANOVA	Analysis of Variance
*aox1*	Alcohol oxidase 1 promoter
BbroAFP	*Beauveria brongniartii* Antifungal Protein
BCA	Bicinchoninic Acid assay
*bctubA*	*Botrytis cinerea* beta-tubulin
BF/WF	Bright-field/Wide-field
BMGY	Buffered glycerol-complex medium
BMM	Buffered minimal methanol
BODIPY	BODIPY-FL-EDA fluorophore
BSA	Buried Surface Area
Caco-2	Human colon adenocarcinoma cell line
CFU	Colony-forming units
CFW	Calcofluor White
CI	Confidence Interval
CLSI	Clinical and Laboratory Standards Inst.
CM-H2DCFDA	5-(and-6)-chloromethyl-2′,7′-dichlorodihydrofluorescein diacetate
DAPI	4′,6-diamidino-2-phenylindole
dpi	Days post-inoculation
EDC/NHS	Coupling chemistry agents
EMEM	Eagle’s minimal essential medium
FBS	Fetal bovine serum
FDR	False discovery rate
HepG2	Human liver cancer cell line
IC_50_	Half-maximal inhibitory conc.
IMAC	Immobilized metal affinity chromatography
ipTM	Inter-chain predicted TM-score
Kd	Dissociation constant
LC–MS/MS	Liquid chromatography–tandem mass spectroscopy
LDH	Lactate dehydrogenase
*mca1/mca2*	Metacaspase 1 and 2
MEA/MEB	Malt extract agar/broth
MIC	Minimum inhibitory concentration
MutS	Methanol utilization slow phenotype
MWCO	Molecular weight cut-off
NaAc	Sodium Acetate buffer
NIS	Non-interacting Surface
Ni^2+^-NTA	nickel-nitrilotriacetic acid
OD_600_	Optical density at 600 nm
PAE	Predicted Alignment Error
RCD	Programmed/Regulated cell death
PCR	Polymerase chain reaction
PI	Propidium iodide
pLDDT	Predicted Local Distance Difference Test
PSM	Peptide spectrum match
pTM	Predicted TM-score
qRT-PCR	Quantitative reverse transcription PCR
RCD	RCD Programmed/Regulated cell death
ROS	Reactive oxygen species
SASA	Solvent-accessible Surface Area
SD/SEM	Standard Deviation/Error of Mean
SDS-PAGE	Sodium dodecyl sulfate-polyacrylamide gel
TUNEL	Terminal deoxynucleotidyl transferase assay
WST-8	Cell viability tetrazolium salt
YPD	Yeast extract peptone dextrose
ΔCt	Cycle threshold difference
ΔG	Predicted binding free energy
