# Supplementary material for: Functional Characterization of BbroAFP Reveals Its Pleiotropic Antifungal Activity in Botrytis cinerea
Source: J Fungi (Basel). 2026 Apr 23;12(5):305. doi: 10.3390/jof12050305 (PMC13207606; doi:10.3390/jof12050305)
Supplement: Supplementary file 1 [file jof-12-00305-s001.zip › jof-4201937-supplementary.pdf]

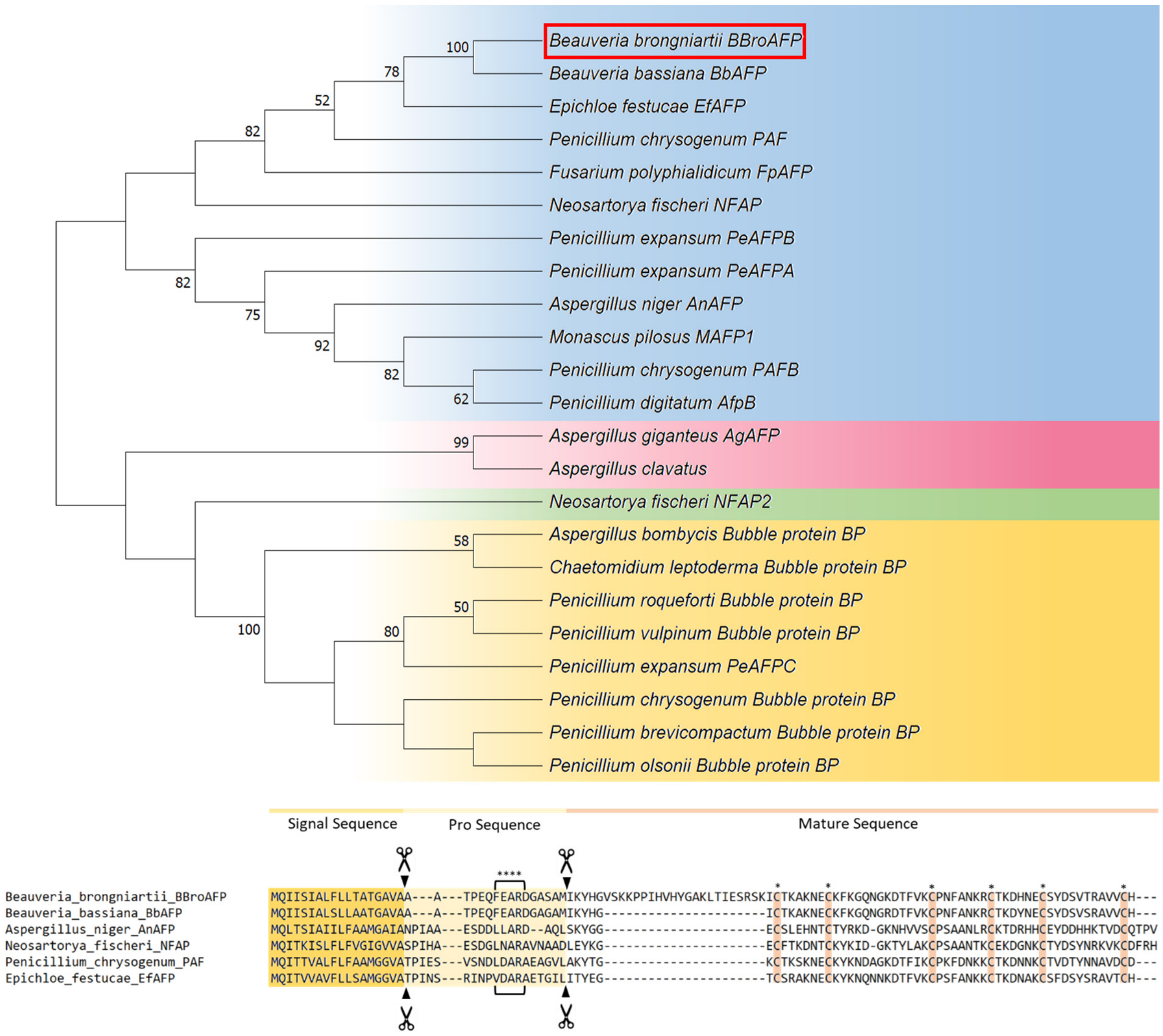


**Figure S1. Phylogenetic and sequence analysis of BbroAFP and related cysteine-rich antifungal proteins.**

(A) Maximum likelihood phylogenetic tree of selected cysteine-rich antifungal proteins (AFPs) constructed based on amino acid sequences using the JTT substitution model with 1000 bootstrap replicates. Bootstrap values ≥50% are shown at the nodes. Phylogenetic analysis was performed using MEGA version 11. Colored background regions indicate major AFP clusters inferred from the phylogenetic analysis. BbroAFP from *Beauveria brongniartii* (highlighted with a red box) clusters with classical PAF-like AFPs (Cluster I), clearly separated from divergent AFPs and bubble protein homologs, which were included as an outgroup. (B) Multiple sequence alignment of representative AFPs highlighting conserved structural features. Signal peptides are indicated in yellow, and predicted signal peptide cleavage sites are marked with scissors. The pro-peptide region is shown immediately downstream of the cleavage site, followed by the mature AFP region. Conserved cysteine residues within the mature peptides are highlighted, illustrating the conserved disulfide bond framework characteristic of PAF-like cysteine-rich antifungal proteins [35].

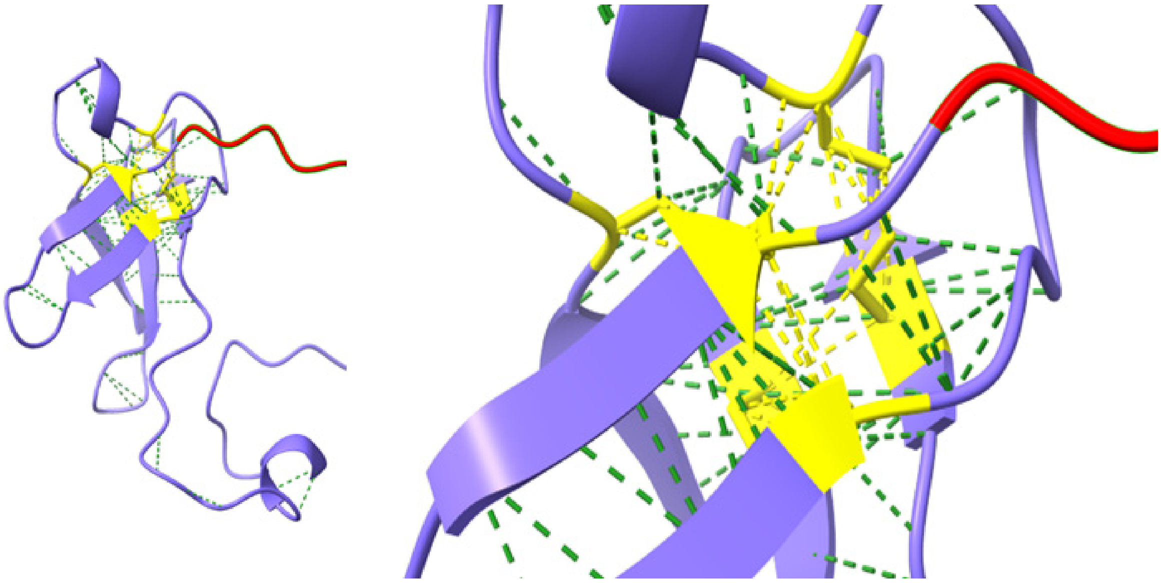

**Figure S2. Predicted structural model of BbroAFP highlighting cysteine interactions.**
The predicted protein structure is shown as a ribbon representation (purple). Cysteine residues are highlighted in yellow, and the C-terminal His-tag is indicated in red. Yellow dashed lines represent distances between cysteine residues consistent with potential disulfide bond formation, while green dashed lines indicate additional intramolecular interactions within the structure. The structural model was predicted using AlphaFold and visualized with UCSF ChimeraX [13,15].

**Table S1. Global Interface Geometry of the Predicted BbroAFP Homodimer:** Interface parameters, including buried surface area and solvent-accessible surface area (SASA), were calculated from the predicted BbroAFP dimer structure. Structural analysis was performed using UCSF ChimeraX. The interface area of 1703 Å² falls within the range typically associated with stable biological homodimers [15].

| Parameter | Value |
|---|---|
| Buried interface area (Å²) | 1703.1 |
| SASA chain A (Å²) | 6727.6 |
| SASA chain B (Å²) | 6733.6 |
| SASA complex (Å²) | 10055.0 |
| Total contacting residues | 82 |
| Interface residues per chain | 41 |
| Interface symmetry | Symmetric |
| Interface classification | Strong biological homodimer (>1500 Å²) |

.

**Table S2. SASA and ΔSASA Comparison Between Monomer and Dimer States:** Solvent-accessible surface area (SASA) values were calculated for the monomeric chains and the predicted dimer complex. ΔSASA represents the reduction in accessible surface area upon dimer formation. Calculations were performed using UCSF ChimeraX, indicating substantial surface burial consistent with stable homodimer formation [15].

| Parameter | Value |
|---|---|
| Mean monomer SASA (Å$^2$) | 6730.6 |
| Combined monomer SASA (A+B) (Å$^2$) | 13461.2 |
| SASA dimer complex (Å$^2$) | 10055.0 |
| Total ΔSASA upon dimerization (Å$^2$) | 3406.2 |
| Buried surface area per monomer (Å$^2$) | 1703.1 |
| % SASA reduction per monomer (Å$^2$) | 25.3% |

**Table S3. Major Interface Residues Ranked by Buried Area (Chain A):** Residues contributing to the BbroAFP dimer interface were ranked based on buried surface area values derived from the predicted structural model. Calculations were performed using UCSF ChimeraX. Hydrophobic and aromatic residues contribute prominently to the interface core, while charged residues contribute to electrostatic stabilization [15].

| Rank | Residue | Buried Area ($Å^2$) |
|---|---|---|
| 1 | LYS19 | 163.04 |
| 2 | TYR16 | 150.17 |
| 3 | VAL70 | 110.07 |
| 4 | ILE12 | 103.17 |
| 5 | TYR3 | 99.24 |
| 6 | GLU23 | 91.16 |
| 7 | LEU20 | 62.37 |
| 8 | HIS15 | 60.95 |
| 9 | SER69 | 57.69 |
| 10 | HIS77 | 57.69 |

**Table S4. Equations of the dimerization analysis in ChimeraX**

| | | |
|---|---|---|
| Equation 1: | Total ΔSASA Upon Dimerization | $\Delta SASA_{total} = (SASA_A + SASA_B) - SASA_{complex}$ |
| Equation 2: | Buried Surface Area Per Monomer | $A_{buried} = \frac{\Delta SASA_{total}}{2} = 1703.1\ \AA^2$ |
| Equation 3: | Percentage SASA Reduction | $\% \, reduction = \frac{A_{buried}}{SASA_{monomer}} \times 100 = 25.3\%$ |

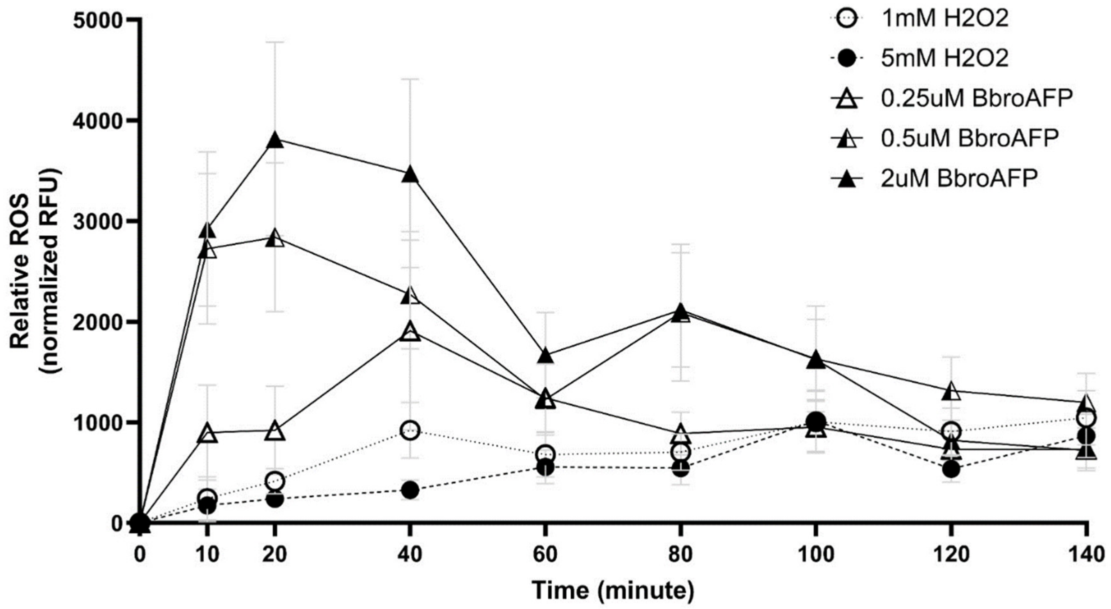


**Figure S3. Extended time-course analysis of intracellular ROS induction following BbroAFP treatment.**

Intracellular reactive oxygen species (ROS) levels were quantified using the fluorescent probe CM-$H_2$DCFDA after treatment with BbroAFP at 0.25, 0.5, and 2 μM, or with 1 mM and 5 mM $H_2O_2$ as oxidative stress controls. Relative ROS fluorescence was monitored over a 140 min time course and normalized to the untreated control at each corresponding time point. BbroAFP induced a rapid and concentration-dependent increase in intracellular ROS, reaching peak levels within the first 20–40 min, followed by a gradual decline at later time points. In contrast, $H_2O_2$ treatment resulted in a slower and more sustained ROS accumulation with lower maximal intensity. Data are presented as mean ± SEM of three technical replicates; the experiment was independently repeated at least three times with comparable trends.

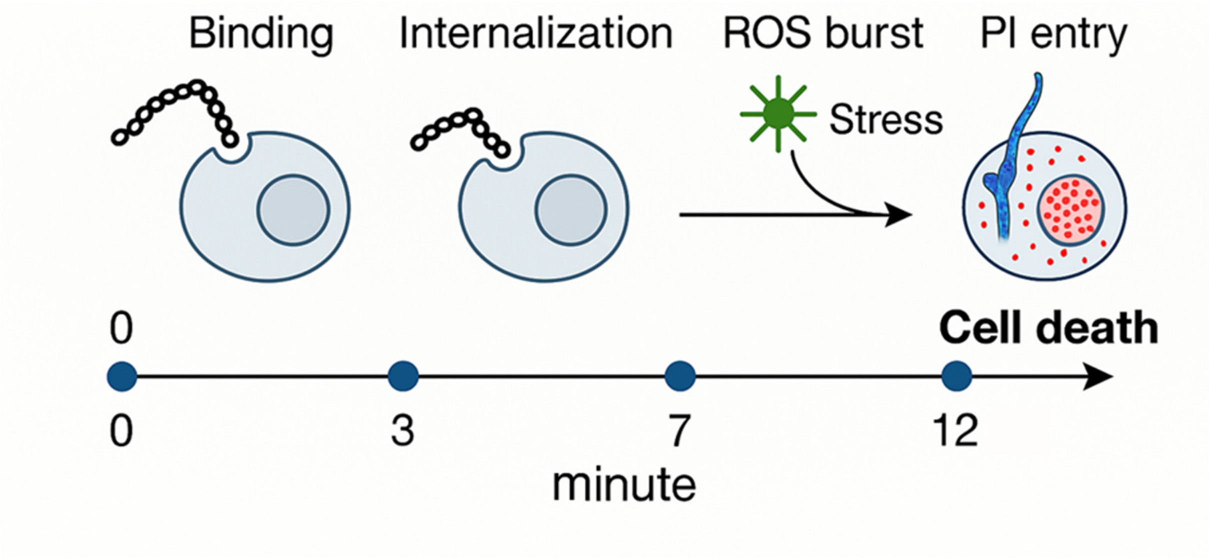


**Figure S4**. **Schematic representation of the proposed mode of action of BbroAFP.**

The model illustrates a time-resolved sequence in which the antifungal peptide initially binds to the fungal cell surface and is subsequently internalized without immediate membrane disruption. Internalization is followed by a rapid intracellular ROS burst, indicating severe cellular stress. At later stages, plasma membrane permeabilization occurs, as evidenced by propidium iodide uptake, suggesting that membrane damage represents a downstream consequence of intracellular stress rather than the primary cause of cell death. The schematic was prepared with the assistance of generative AI tools and manually edited for scientific accuracy.

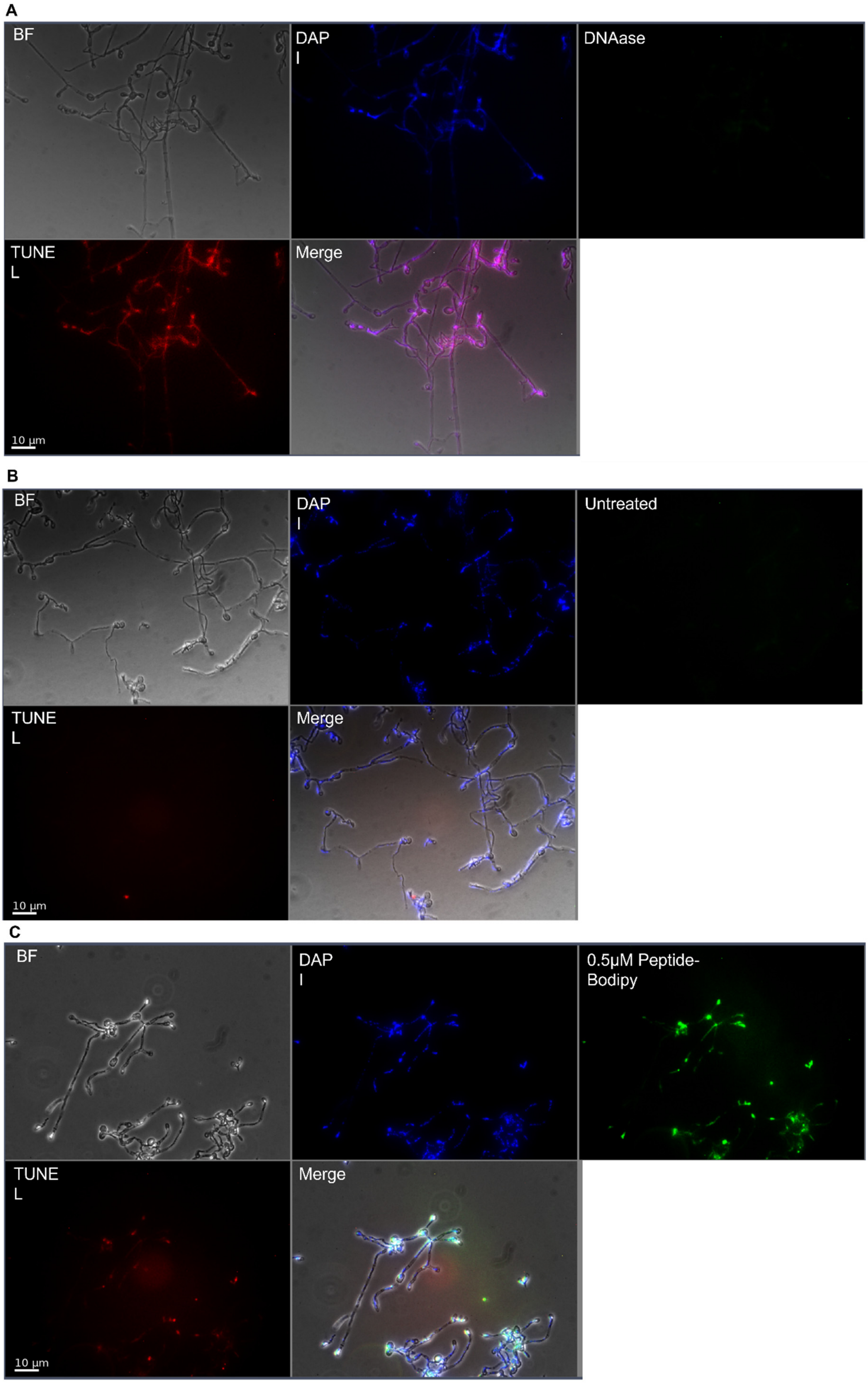

A
BF
DAP
I
DNAase
TUNE
L
Merge
10 µm
B
BF
DAP
I
Untreated
TUNE
L
Merge
10 µm
C
BF
DAP
I
0.5µM Peptide-
Bodipy
TUNE
L
Merge
10 µm

**Figure S5. Validation of TUNEL assay specificity and evidence of BbroAFP-associated nuclear DNA damage in *Botrytis cinerea* hyphae.**

Representative wide-field fluorescence microscopy images showing control and peptide-treated conditions. Panels include bright-field (WF), nuclear staining (DAPI, blue), TUNEL staining (red), peptide localization (BODIPY, green), and merged images. (A) DNase I–treated hyphae (positive control) exhibit strong and widespread TUNEL fluorescence (red), confirming efficient labeling of DNA strand breaks and demonstrating the sensitivity and functionality of the TUNEL assay under the experimental conditions. DAPI staining reveals nuclear structures, while the absence of BODIPY signal confirms that no peptide is present in this control. (B) Untreated control hyphae show intact hyphal morphology in bright-field images and clearly defined nuclei (DAPI, blue), with no detectable TUNEL signal. This confirms the absence of basal DNA fragmentation and validates the specificity of the TUNEL assay in non-stressed cells. (C) Hyphae treated with BbroAFP (0.5 μM) display distinct TUNEL-positive signals (red), indicating DNA strand breaks. These signals spatially overlap with DAPI-stained nuclei, suggesting that DNA damage occurs at the nuclear level. BODIPY-labeled BbroAFP (green) is detected within the same cells, indicating intracellular peptide accumulation. The merged images illustrate the co-occurrence of peptide presence and DNA fragmentation within individual hyphae. While the spatial overlap of TUNEL and DAPI signals supports nuclear DNA damage, it should be noted that the resolution of wide-field fluorescence microscopy does not allow precise assignment to specific subnuclear compartments or definitive co-localization at the molecular level. Scale bar: 10 μm.

**Table S5.** The technical validity of the qPCR assays was confirmed via melt curve analysis across three biological replicates. All target genes exhibited high technical reproducibility (>99.0%) and consistent melting temperatures (Tm), with no evidence of primer-dimer formation.

| **Target Gene** | **Melting Temp (Tm)** | **Technical Reproducibility** | **Peak Specificity** |
|---|---|---|---|
| ***bctubA*** | 81.21 ± 0,15 °C | **>99.8%** | Single, Sharp |
| ***bcmca1*** | 84.51 ± 0,12 °C | **>99.8%** | Single, Sharp |
| ***bcaif1*** | 79.62 ± 0,28 °C | **>99.6%** | Single, Sharp |
| ***bcmca2*** | 80.22 ± 0,46 °C | **>98.6%** | Single |

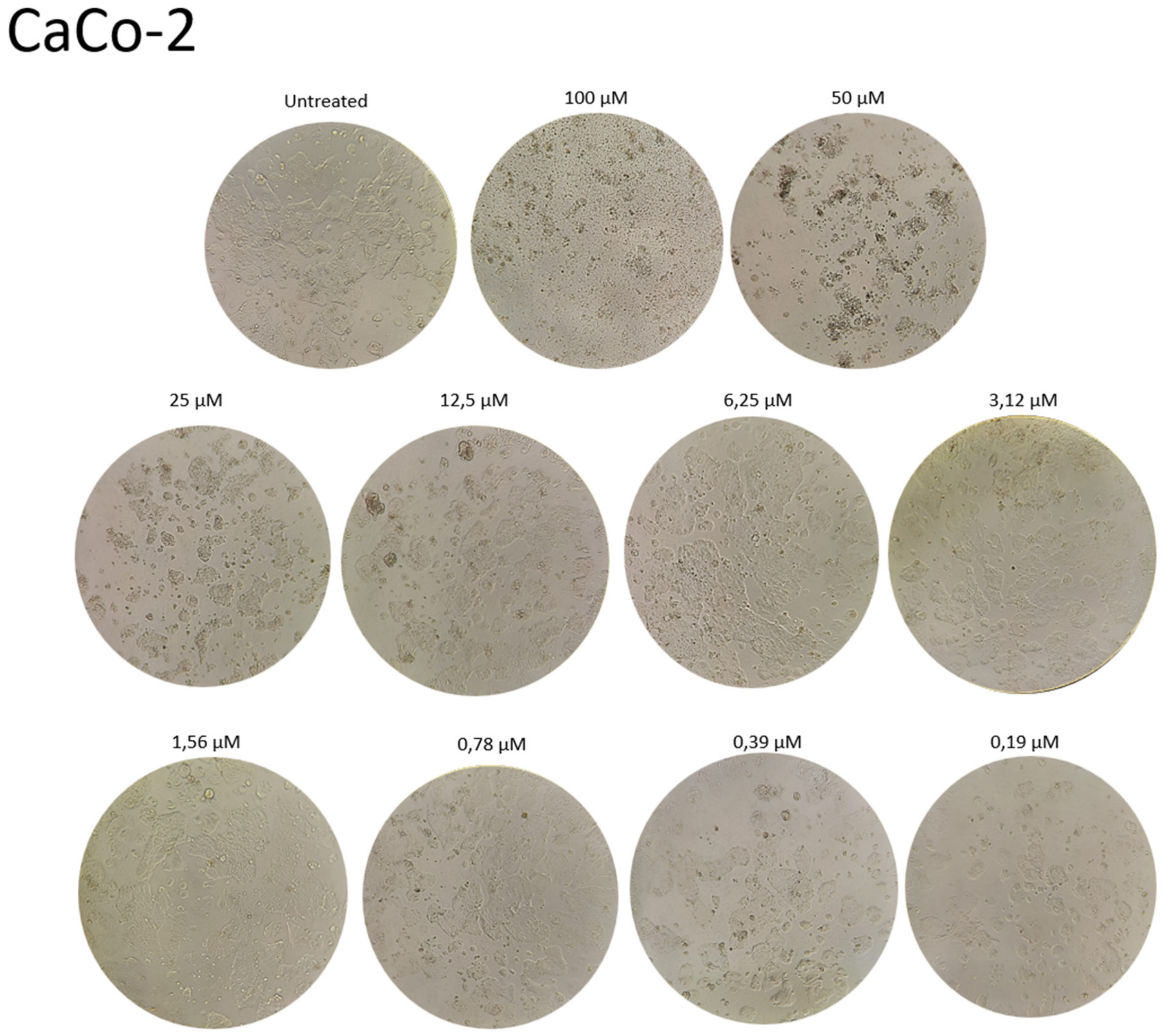


**Figure S6. Phase-contrast microscopy-based morphological evaluation of Caco-2 cells following 24 h exposure to ten different concentrations of the BbroAFP protein.** At low and intermediate concentrations, the overall cellular morphology was largely preserved, with intact cell–cell contacts and maintained adhesion to the culture surface. In contrast, treatment with higher concentrations resulted in pronounced morphological alterations, including reduced cell density, increased cellular rounding, and a tendency of cells to detach from the substrate. These observations are consistent with the quantitative cytotoxicity assays. Images were acquired using an inverted phase-contrast microscope (×20 objective) with digital capture via a smartphone camera adapted to the eyepiece.

# HepG2

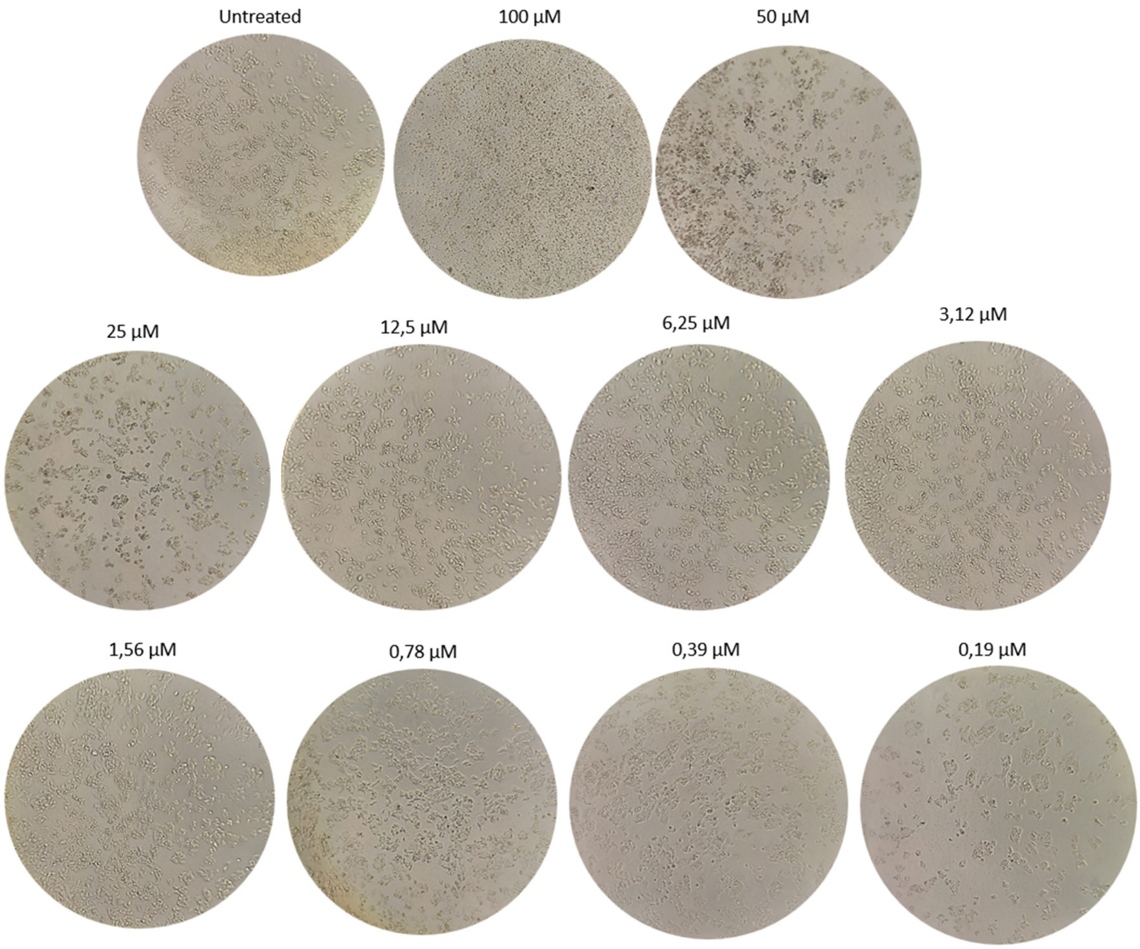


**Figure S7. Phase-contrast microscopy-based morphological evaluation of HepG2 cells following 24 h exposure to ten different concentrations of the BbroAFP protein**. At low and intermediate concentrations, the overall cellular morphology was largely preserved, with intact cell–cell contacts and maintained adhesion to the culture surface. In contrast, treatment with higher concentrations resulted in pronounced morphological alterations, including reduced cell density, increased cellular rounding, and a tendency of cells to detach from the substrate. These observations are consistent with the quantitative cytotoxicity assays, suggesting a dose-dependent but non-abrupt cytotoxic response in hepatic cells. Images were acquired using an inverted phase-contrast microscope (×20 objective) with digital capture via a smartphone camera adapted to the eyepiece.
